# A Technology-Supported Guidance Model to Increase the Flexibility, Quality, and Efficiency of Nursing Education in Clinical Practice in Norway: Development Study of the TOPP-N Application Prototype

**DOI:** 10.2196/44101

**Published:** 2023-02-03

**Authors:** Andréa Aparecida Gonçalves Nes, Jaroslav Zlamal, Silje Christin Wang Linnerud, Simen A Steindal, Marianne Trygg Solberg

**Affiliations:** 1 Lovisenberg Diaconal University College Oslo Norway; 2 Institute of Nursing Faculty of Health Studies VID Specialized University Oslo Norway

**Keywords:** clinical practice, guidance application model, nursing students, constructive alignment, metacognition, technological tool, nursing, nursing profession, application, mobile health, eHealth, educator, communication

## Abstract

**Background:**

The challenges of nursing shortage in the nursing profession and of limited nursing educational capacity in nursing education in clinical practice need to be addressed to ensure supply according to the demand of these professionals. In addition, communication problems among nursing students, nurse educators, and nurse preceptors; variations in the guidance competence of nurse preceptors; and limited overview from nurse educators on nursing students’ clinical practice are common challenges reported in several research studies. These challenges affect the quality of nursing education in clinical practice, and even though these problems have been highlighted for several years, a recent study showed that these problems are increasing. Thus, an approach is required to ensure the quality of nursing education in clinical practice.

**Objective:**

We aimed to develop a guidance and assessment application to meet the challenges reported in clinical practice. The application intended to increase the flexibility, quality, and efficiency of nursing education in clinical practice. Furthermore, it intended to increase interactive communication that supports guidance and ensure structured evaluation of nursing students in clinical practice.

**Methods:**

This study employed a multidisciplinary user-participatory design. Overall, 23 stakeholders from the project team (ie, 5 researchers, 2 software developers, 1 pedagogical advisor, and 15 user representatives [4 educators, 6 preceptors, and 5 students]) participated in a user-centered development process that included workshops, intervention content development, and prototype testing.

**Results:**

This study resulted in the creation of the Technology-Optimized Practice Process in Nursing (TOPP-N) guidance and assessment application for use as a supportive tool for nursing students, nurse preceptors, and nurse educators in clinical practice. The development process included the application’s name and logo, technical architecture, guidance and assessment module, and security and privacy.

**Conclusions:**

This study offers insights into the development of an evidence-based technological tool to support nursing students, nurse preceptors, and nurse educators in clinical practice. Furthermore, the developed application has the potential to meet several challenges reported in nursing education in clinical practice. After a rigorous development process, we believe that the TOPP-N guidance and assessment application prototype is now ready to be tested in further intervention studies.

## Introduction

### Background

The need for registered nurses (RNs) is increasing worldwide [1,2], and this has been made clear by the advent of the COVID-19 pandemic [3]. In Norway, a shortage of 28,000 nurses is predicted by 2035 [4]. To meet the future demand for RNs, strategies are needed to increase educational capacity and the number of nursing students (NSs) completing the bachelor’s program in regular time with ensured educational quality.

In line with European Union directives [5,6], 50% of the Norwegian nursing education program consists of clinical practice, and due to educational logistics (ie, to avoid displacing educators and students), it is recommended that clinical placements be near educational institutions. This limits educational capacity, because several clinical placements that are far from educational institutions are not being used. Another challenge in nursing education is the current guidance model used in many educational institutions in European countries, including Norway, which cannot ensure that NSs achieve the learning outcomes and the expected quality of clinical practice [7-9]. Therefore, a strategy is urgently needed to increase the number of eligible clinical placements and ensure the learning quality of NSs and their achievement of expected learning outcomes in clinical education.

### Challenges Related to the Norwegian Guidance Model

The current Norwegian guidance model for clinical practice involves the following 3 parties: an NS, a nurse preceptor (NP), and a nurse educator (NE). The NP is a RN employed by the health care institution where the NS is present during the clinical practice period (eg, nursing home and hospital), and the NE is employed by an educational institution (ie, the university or university college where the NS receives nursing education). The NP role involves daily face-to-face guidance, follow-up, supervision, and evaluation of the NS. The NE is responsible for ensuring that the clinical practice period provides the NS with optimal learning and a fair assessment of the achieved learning outcomes. This guidance model for clinical practice is described in detail elsewhere [10].

Users experience the current guidance model as fragmented, and it leads to many challenges in clinical practice [7]. A guidance culture and environment are important for NSs to achieve learning outcomes in practical studies [11], but NSs report feelings of isolation and limited cooperation with their NEs and peers [8,12,13]. Furthermore, NSs work with NPs of varying pedagogical competence in clinical guidance, which directly impacts the achievement of their learning outcomes [14].

Both NPs and NSs stress that NPs need to develop their pedagogical competence [7,15]. Thus, a key to overcoming the challenges in clinical practice is to increase NPs’ pedagogical competence and, consequently, guidance skills [16]. Meanwhile, NPs report that insufficient time is the greatest challenge in guiding NSs in clinical practice, as time-consuming preceptorship must be conducted in addition to all the responsibilities and duties of an RN. NEs also find that the current practice model supports only limited contact among NEs, NSs, and NPs, which could result in insufficient oversight of what is happening in clinical practice. Research shows that when a challenge occurs in clinical practice, the NS and NP wait too long to involve the NE, making it harder to solve challenges at an early stage [7]. Although the above-mentioned challenges have been underlined for many years, a recent research study suggested that the challenges related to clinical practice in nursing education have only increased in the past 10 years [17].

Moreover, due to the increasing complexity of modern health care demands [18], critical thinking is a desired outcome in nursing education. Defined as the process of making a reflective judgment [19] about what to believe or do in a given context [20], critical thinking is needed to acquire the expected competence in clinical practice in nursing education.

Communication is the main element of instruction, and digital communication has developed greatly in recent years, facilitating active and remote learning (eg, the use of digital solutions during the COVID-19 pandemic) [21]. Wireless devices, such as smartphones, tablets, and computers, have become an integral part of society and provide access to necessary information and educational tools independent of physical location [22,23]. These devices and the available technology provided the necessary structure for the development of an application aimed at improving communication among NSs, NPs, and NEs in guidance, mitigating the challenges of nursing education in clinical practice.

When developing a technological tool, such as a guidance application, it is recommended that the developers follow an approach that involves all stakeholders (in this case, NSs, NPs, NEs, researchers with expertise in the explored field, and information technology designers and developers) from the early stage (idea generation) to the final stage (evaluation and implementation). However, few studies have followed this recommendation [24]. Furthermore, a recent mixed methods review showed that technological tools tailored to support guidance of NSs in clinical practice that meet the challenges faced by Norwegian educational institutions and stimulate students’ critical thinking are missing [18].

### Objective

This study aimed to develop a guidance and assessment application to meet the challenges in the nursing profession and education; improve communication; support guidance; stimulate NSs’ critical thinking; ensure structured evaluation of NSs in clinical practice; and increase the flexibility, quality, and efficiency of nursing education.

## Methods

### Study Design

This study was performed between September 2019 and March 2020, and employed a multidisciplinary user-participatory design approach [25,26] to ensure that the developed application would be acceptable (ie, well received, suitable, user friendly, and attractive) and designed to fit the needs of users (NSs, NPs, and NEs) and the context of use. The study is part of a larger project to develop, test the effectiveness of, and implement technology-supported guidance to increase the flexibility, quality, and efficiency of nursing education in clinical practice. The study protocol was published prior to the development of the application [10]. The usability, feasibility, and effect of the developed application have been tested, and the results will be presented in future papers.

### Recruitment

NSs were recruited through study information published on Canvas (Instructure Inc), a learning management system used by Lovisenberg Diaconal University College (LDUC), while NPs were recruited through an existing cooperation agreement among LDUC, a selected nursing home, and a university hospital in Oslo. NEs were invited to participate in the study by members of the project group. To be eligible to participate in the development of the application, researchers had to be members of the project group, NSs had to have experience of at least one clinical practice, and NPs and NEs had to have experience of guiding NSs in clinical practice. It was important for user representatives to have experience in primary and secondary health care.

### Sample

The application development was led by the principal investigator (AAGN), who is an associate professor with long experience as an RN and NE employing active learning methods. The multidisciplinary project group comprised researchers in nursing education with experience of clinical practice mentoring, who also performed the role of NEs (1 doctoral student, 2 associate professors, and 2 professors). Additionally, 1 application developer and 1 designer (from MOSO, a company that cooperated in developing the application), as well as 1 pedagogical advisor contributed to the application development. The members of the project group included 4 NEs representing all the year units in nursing education, 6 NPs representing clinical practice in primary and secondary health care, and 5 NSs in their second or third year of education (Table 1). The researchers, NEs, NSs, and pedagogical advisor were from LDUC.

**Table 1:** Members of the project group during the development process.

Members and competence/qualification		Value (N=23), n
**Researcher**		5
	Assistant professor	1
	Associate professor	2
	Professor	2
**Nurse educator**		4
	Assistant professor	4
**Pedagogical advisor**		1
	Assistant professor	1
**Nurse preceptor**		6
	Primary health care	2
	Secondary health care	4
**Nursing student**		5
	Second study year	3
	Third study year	2
**Application developer**		2
	Function	1
	Design	1

### Ethics Approval

The study was approved by the Institutional Research Board at LDUC and the Norwegian Centre for Research Data (reference number: 338576). Each participant was informed that the meetings would be documented in minutes and the minutes would be used as data to be analyzed in this study. After receiving the information, the participants provided oral informed consent to participate in the development process of the application.

### Application Development

The starting point in the development of the application was a concept adopted from a health care intervention created by the principal investigator (AAGN), in which patients filled out digital electronic reports (e-reports)/diaries and received situational feedback from an RN regarding management of their diabetes [27]. The idea was that the concept of writing e-reports on one’s learning and the concept of communication between users could be transferred to and further developed into a guidance application for use in clinical practice in nursing education.

Before the development started, a contextual inquiry collected information on users’ needs and the requirements for acceptability in nursing homes and hospital units [25]. Target users were involved to elucidate the challenges related to clinical practice from the perspectives of NSs, NPs, and NEs. The development process also used the results of earlier studies on the challenges in guiding NSs in clinical education [12,17,28,29].

#### Project Management Based on the Spiral Model of Application Development

The spiral model, an approach to software development that uses an adaptive, incremental, and iterative working method in organized multidisciplinary groups, was chosen due to the need for flexibility. This model focuses on addressing risks and involving users in the development process. The project manager is responsible for generating a win-win outcome for users, customers, group members, and concerned stakeholders [30].

**Figure 1:**
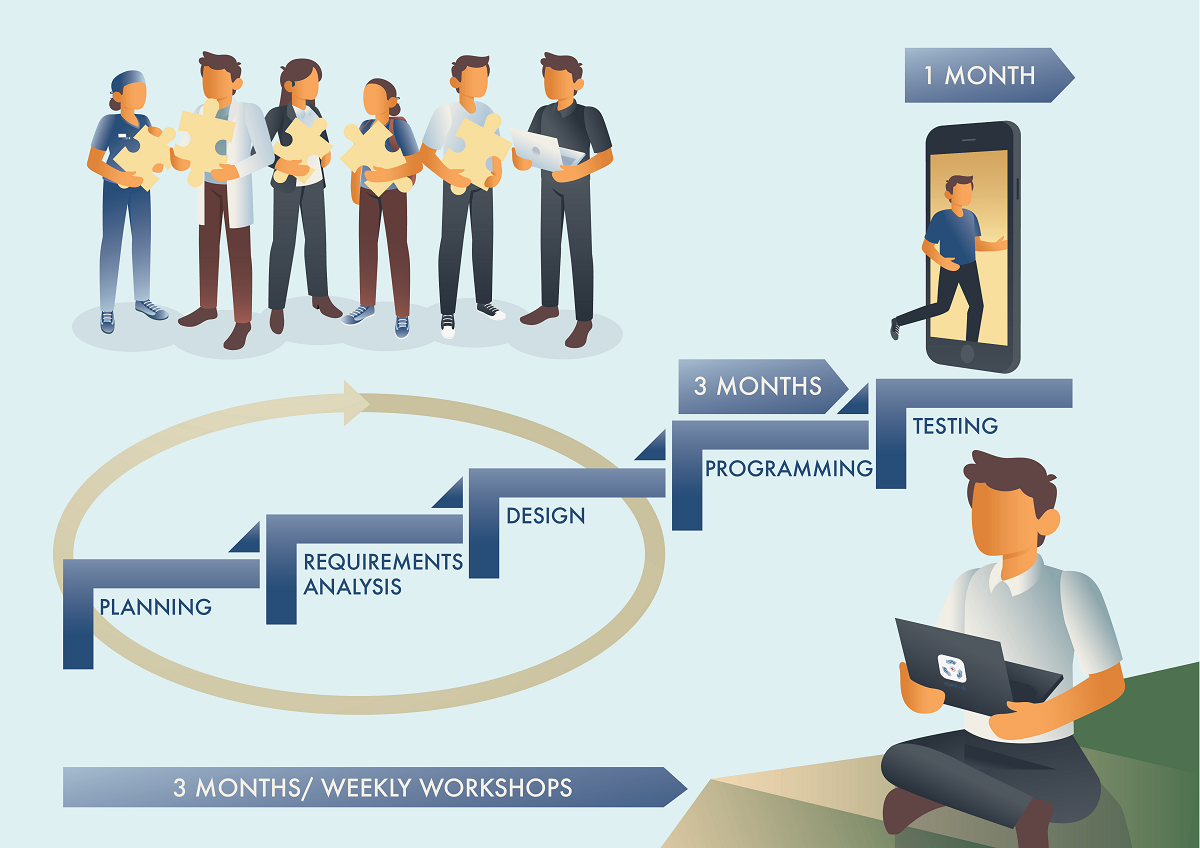
The application development process.

The application development process involved the following phases: (1) planning, (2) requirement analysis, (3) design, (4) programming, and (5) testing. In the first 3 phases, weekly workshops were held to discuss the application’s function, content, and process, which were evaluated with the application developers before decisions were made about application functionality (Figure 1). The programming was done by the developers and tested by the user representatives.

#### Workshops

As part of the contextual inquiry, stakeholders were invited to a daylong workshop to discuss their needs, ideas, requirements for acceptability, and the potential challenges related to the application’s guidance element. Next, we held weekly 2-hour workshops for 3 months. In the first 2 months, the stakeholders were divided into 3 smaller multidisciplinary groups consisting of 1 researcher and at least one NS, one NP, and one NE. Group discussions lasted 1 hour and were led by the researcher. In the next hour, the whole project group met the application developer and application designer to discuss ideas related to content, functions, and design. We pursued an inductive process in which users could suggest ideas on design and content features to further explore users’ requirements for the application.

After the second workshop, the project leader changed the strategy of the work process. Based on input from previous meetings and the mandatory elements of the application’s content and functionality, a draft of the potential design feature, created by AAGN and JZ, was presented to the project group. The mandatory content was based on the NSs’ assessment criteria and their expected learning outcomes in clinical practice. The application’s functions aimed to stimulate critical thinking and promote the achievement of the learning outcomes. After the presentation, AAGN and JZ asked the project group members to give their opinions and suggestions for changes and improvements. In each workshop, a topic related to content, functionality, design, or barriers to use was presented and discussed. All the workshops were facilitated by the project leader (first author) in collaboration with other project group members (JZ, MTS, and SAS). Notes were taken on each group discussion.

#### Development of the Application’s Name and Promotional Material

The development of an application is regarded as the development of a new product [31] that requires a brand name. A brand’s primary role is to convey awareness and a favorable impression of the product [32]. An application’s icon should effectively convey meaning, easily communicate the intended function of the application, and enable the user to create associations and meaning [33]. Ideas for the application’s name, logo, icon, and promotional materials were drafted by JZ and AAGN, and then completed by a dedicated graphic designer. The draft was presented to the project group for suggestions, adjustments, and approval.

#### Application Functions Based on Constructive Alignment and the Concept of Metacognition

The constructive alignment principle [34] was adopted in building the application’s learning functions to ensure a connection between learning outcomes, learning activities, and the assessment of clinical practice. The following 3 main phases of the metacognitive cycle were applied: (1) planning and goal setting, (2) applying strategies and monitoring progress, and (3) evaluating and adapting approaches [35]. Metacognition plays a crucial role in the development of critical thinking, so we aimed to support NSs in achieving the clinical practice’s learning outcomes and developing their critical thinking, as RNs who are competent in critical thinking are better prepared to meet the constantly changing and developing challenges of health care [36].

#### Application Content Based on Learning Outcomes and Assessment Criteria

In nursing education, it is commonly challenging to ensure that the learning outcomes for clinical practice have been achieved, and several approaches to assessment have been tried [37-39]. The developed guidance and assessment application employs a validated research-based assessment instrument, the Assessment of Clinical Education (AssCE) [40], whose copyright holders approved its use. The AssCE was developed in Sweden, has been used since 1999, and was originally developed under the general guidelines for Swedish and international requirements [40]. It assesses NSs’ expected learning outcomes during the entire education period.

### Data Collection and Analysis

The data included recorded notes from the workshops that were initially summarized by the first author, who used rapid analysis [41] to ensure that the material provided essential input for the ongoing development. Rapid approaches to collecting and analyzing data can accelerate an application’s development while maintaining scientific rigor [42]. The data were extracted and compared across the various workshops to identify similarities and differences in the material. Based on the results of constant data analysis, a requirement specification was elaborated for the application, and a prototype was developed.

## Results

### Overview

The results presented in this article are limited to the application prototype, including the guidance and assessment module, technical architecture, security, and privacy.

### Application Prototype

**Figure 2:**
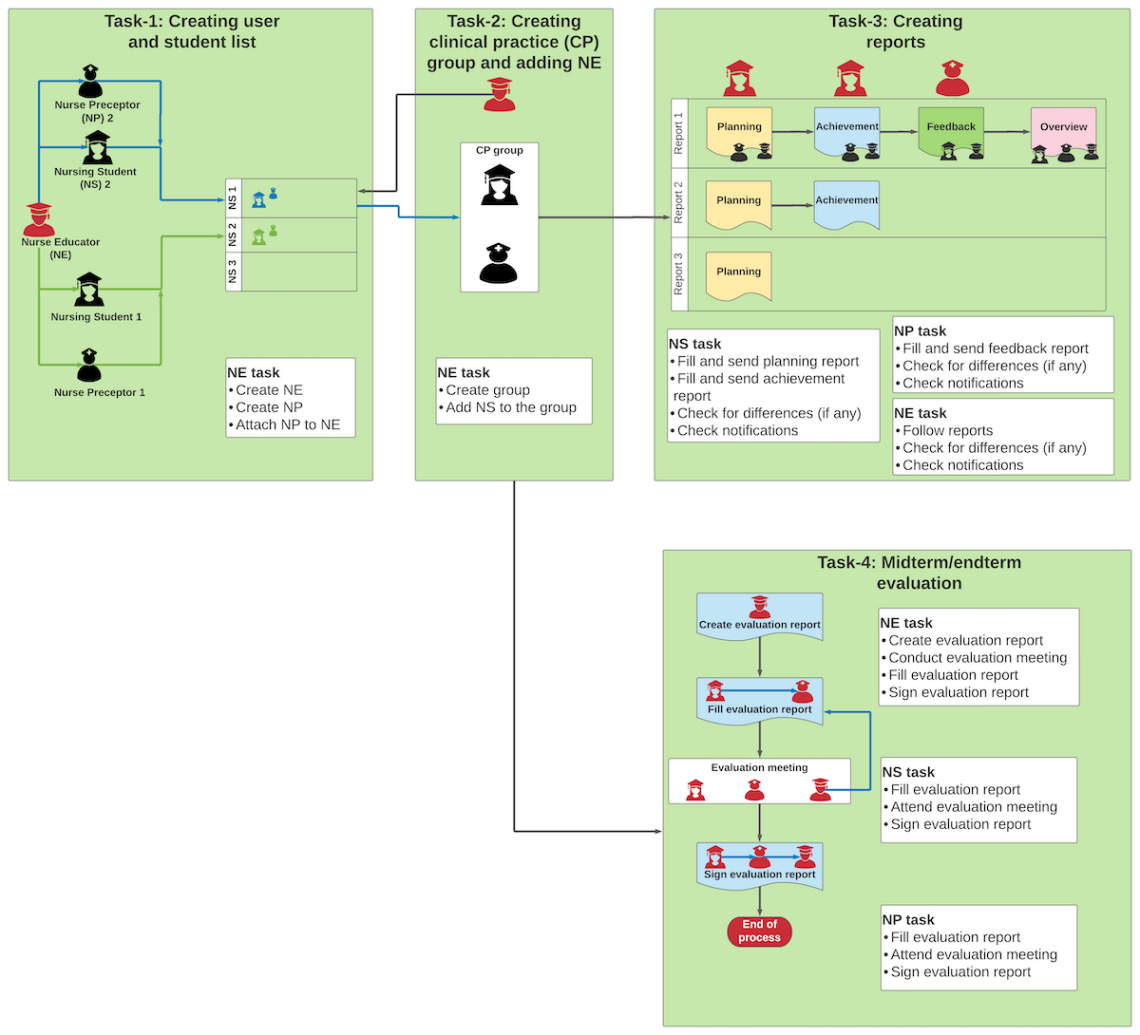
Workflow of the application prototype. NE: nurse educator; NP: nurse preceptor; NS: nursing student.

The developed application prototype was intended to make clinical practice studies more flexible through improved communication, a structured evaluation of NSs, and better integration among NPs, NEs, and NSs. Figure 2 shows the workflow processes of the developed application.

#### Development of the Application’s Name and Promotional Material

The name chosen for the developed application was Technology-Optimized Practice Process in Nursing (TOPP-N). The initial name concept was presented to the project group. The rationale behind the name was that it should highlight the application’s functions and offer a symbol to which a positive meaning could be attributed. In our case, the word *topp* in Norwegian means “being the best, being on the top” [43]. In English, the equivalent written word is *top* [44], which phonetically conveys the same meaning as the Norwegian term. The meaning of “being the best, being on the top” also informed the application’s promotional material, including a poster that shows 3 individuals (NS, NE, and NP) holding digital devices while on their way to the “top,” symbolized as a mountain’s summit (Multimedia Appendix 1). The application’s name also denotes that learning in nursing is a process that, in this case, is supported by technology. In developing the visual elements, such as the application’s icon, we focused on reflecting the meaning of *top*.

The initial concept of the application’s name was accepted by the project group, but several changes were made in the process of developing the icon. When the first idea for the icon was presented to the project group (Multimedia Appendix 2), the group reacted negatively, saying that the icon indicated a “tour planning application” or “hiking application” rather than a guidance application for NSs. The icon was further conceptualized by JZ and AAGN, and a second idea was presented to the group (Multimedia Appendix 3). This time, the initial goal was to symbolize the application’s functions of cooperation and communication, conceptualized as 3 interacting hands. A Facebook poll was created to choose the most liked icon, and the winning draft icon was then submitted to another designer, who created a final version (Multimedia Appendix 4) that shows 3 hands (representing the application’s stakeholders: NSs, NEs, and NPs) surrounding a digital button (representing technology).

#### Guidance Module

**Figure 3:**
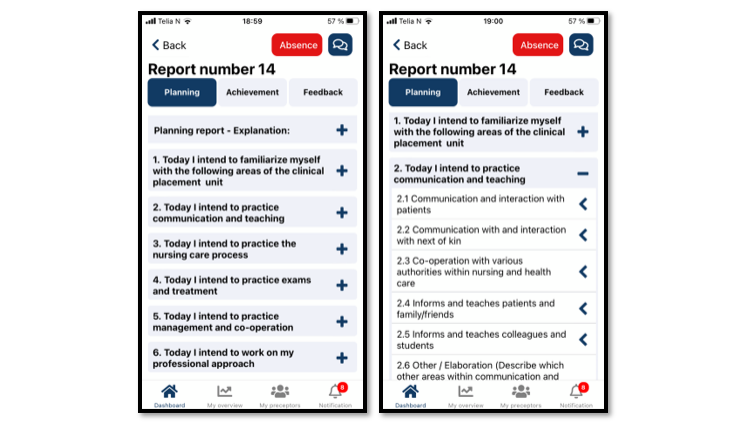
Example of a TOPP-N planning report (mobile version screenshot). TOPP-N: Technology-Optimized Practice Process in Nursing.

**Figure 4:**
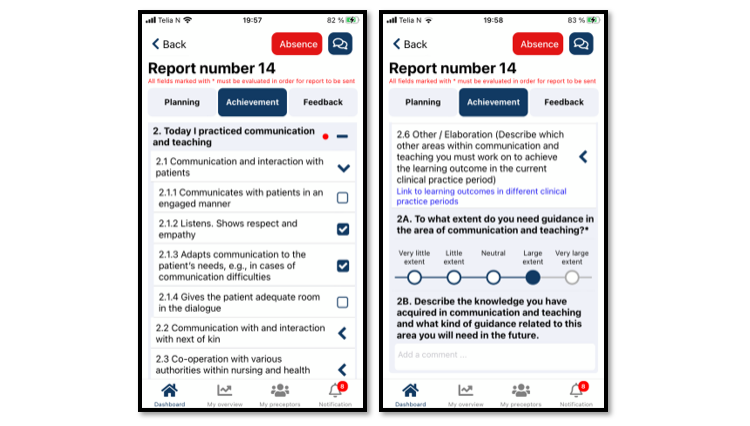
Example of a TOPP-N achievement report (mobile version screenshot). TOPP-N: Technology-Optimized Practice Process in Nursing.

The TOPP-N guidance module’s main functions include NSs’ e-reports and NPs’ feedback to NSs. By completing e-reports, NSs establish and plan their learning goals, document their experience in clinical practice, and monitor their progress toward their learning outcomes. The e-reports include a planning report (to be completed before the start of the clinical practice day; see Figure 3) and an achievement report (to be completed at the end of the day; see Figure 4). The NSs can evaluate and adapt their approaches on the basis of ongoing experience, learning in clinical practice, and tailored feedback from NPs. The e-reports employ a multiple choice format based on the AssCE [40]. A text field is also available, making it possible to describe and explain a reported situation as necessary (see Textbox 1, point 2.6).

**Application content in the area of communication competence.**

2. TODAY, I HAVE AS A PLAN TO PRACTICE COMMUNICATION

- 2.1 Communication and interaction with patients
  2.1.1 Communicates with patients in an engaged manner.
  2.1.2 Listens; shows respect and empathy.
  2.1.3 Adapts communication to the patient’s needs, for example, in cases of communication difficulties.
  2.1.4. Gives the patient adequate room in the dialogue.
- 2.2 Communication with and interaction with next of kin
  2.2.1 Communicates with and listens to the viewpoints of family/friends.
  2.2.2 Shows respect and empathy.
  2.2.3 Creates a dialogue with family/friends and treats their viewpoints with respect.
- 2.3 Cooperation with various authorities within nursing and health care
  2.3.1 Communicates, consults, and confers with others.
  2.3.2 Ensures continuity in the patient’s chain of care.
  2.3.3 Collects, discusses, and critically evaluates relevant information with various authorities and cooperates to ensure appropriate patient care.
  2.3.4 Provides correct information to appropriate authorities.
- 2.4 Informs and teaches patients and family/friends
  2.4.1 Identifies individual needs.
  2.4.2 Organizes and carries out planned instructions.
  2.4.3 Adapts information and instructions for self-care.
  2.4.4 Provides health-promoting and preventive advice and support.
  2.4.5 Follows up on understanding.
  2.4.6. Ensures that the patient and family/friends receive coordinated and continuous information and instructions based on their needs and wishes.
  2.4.7. Uses various aids and techniques creatively.
- 2.5 Informs and teaches colleagues and students
  2.5.1 Demonstrates the ability to seek out and convey information on the patient, situation, and care problems.
  2.5.2 Describes his/her own intended educational outcomes.
  2.5.3 Teaches and supervises upper secondary students, classmates, or equivalent students.
  2.5.4 Critically evaluates information concerning various care issues and conveys it in an interesting manner.
  2.5.5 Teaches and supervises with a view to facilitate development and knowledge growth.
- 2.6 Other (Describe which other areas within communication you must work on to achieve the learning outcome related to the current practice period) – Link to the learning outcome for communication for various practice periods

Seek out learning situations in which communication can be challenging, such as patients with impaired language, hearing, another mother tongue, etc.

**Figure 5:**
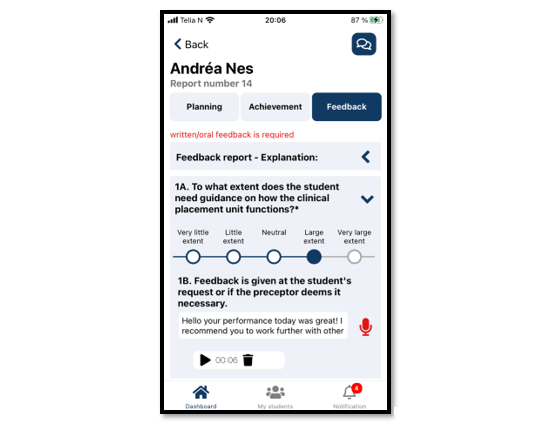
Example of nurse preceptor feedback (mobile version screenshot).

With regard to the metacognition cycle, completing the planning report increases NSs’ awareness of the learning outcomes and their ability to focus on activities that contribute to achieving them. Completing the achievement report stimulates students to reflect on what they learned that day. The NSs’ e-reports are stored on a secure server administered by MOSO, to which NPs have immediate access. Thus, NPs can enter the metacognitive cycle at any stage through tailored feedback or guidance that contributes to NSs’ development. NPs’ feedback to NSs is informed by the e-reports and the preceptors’ daily experience of NSs and may be delivered either in writing or verbally through an audio file available through the application. NSs and NPs can also communicate directly with each other by text message. This process gives NSs the opportunity to adjust their plans and activities. To ensure that they share a common understanding of guidance needs and expectations, NSs and NPs must evaluate each generated report using a 5-point Likert scale covering the NSs’ need for guidance in the 6 distinct nursing competence areas shown in the guidance module (Figures 4 and 5).

**Figure 6:**
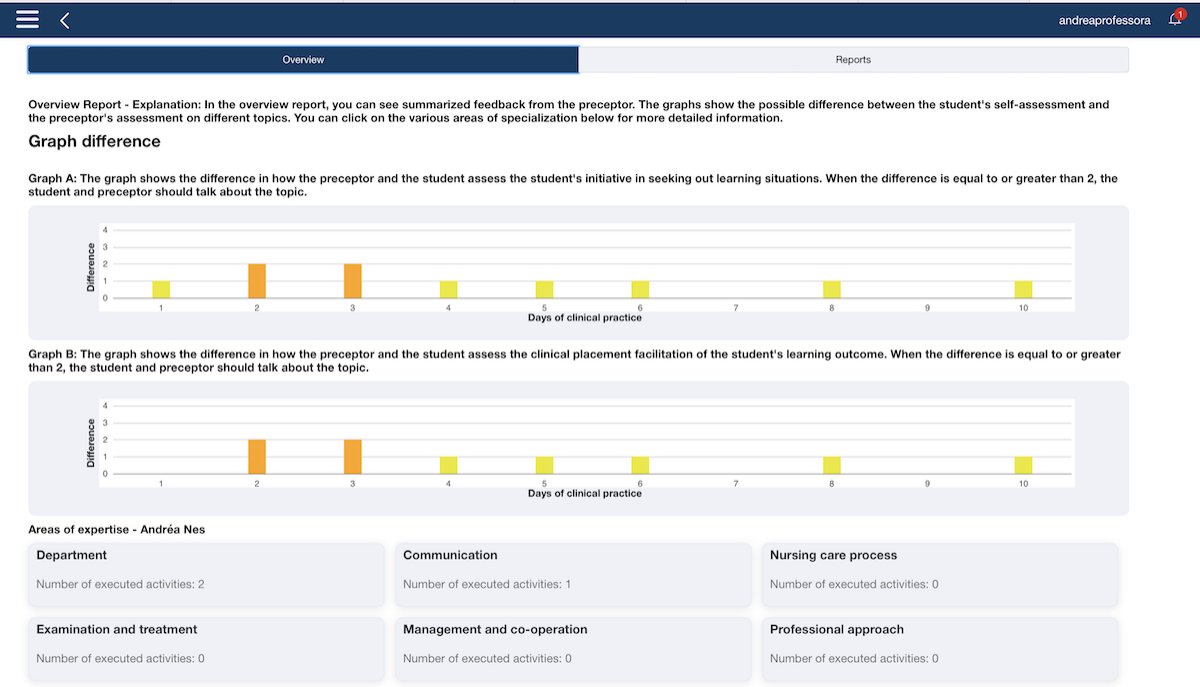
Two graphs revealing disagreement between a nursing student and a nurse preceptor related to evaluation of the student’s need for supervision (web version screenshot).

NEs can access reports and feedback overviews at any time (Figure 6) by logging into TOPP-N and can support NSs and NPs when necessary. Consequently, the supervision during clinical practice is well documented, providing a better basis for assessing NSs.

#### Assessment Module

**Figure 7:**
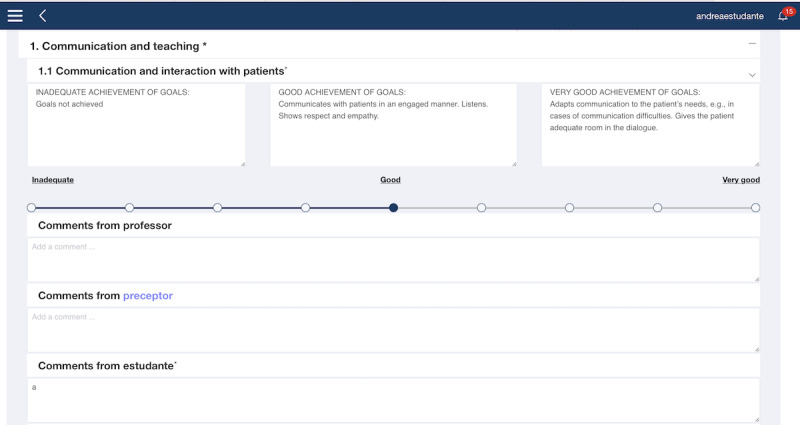
Example of an assessment point in the Assessment of Clinical Education with grading and explanatory text (web version screenshot).

The assessment module (Figure 7) is a digitalization of the AssCE with 21 assessment points under the following 5 main headings: (1) communication and teaching, (2) the nursing process, (3) examinations and treatments, (4) management and cooperation, and (5) professional approach. Each assessment point includes a visual analog scale to identify the target level achieved during clinical practice. The assessment points are also accompanied by explanatory text that corresponds to the 3 levels of goal achievement: “inadequate achievement of goals,” “good achievement of goals,” and “very good achievement of goals.” Detailed information on the AssCE can be found elsewhere [40]. Digitalizing the AssCE enabled NSs, NPs, and NEs to prepare in advance for evaluation meetings and offered flexibility in conducting the meetings (remotely or in person).

The participation of user representatives was essential for achieving the presented results. They stressed that the application had to accommodate NSs’ varied education levels and respective challenges. Moreover, they emphasized that the application should focus not on daily evaluation but on daily guidance and should offer documentation for a fair summative and formative evaluation of clinical practice. NSs sought input on their daily activities through a solution that offered useful, effective, and personalized advice on learning outcomes. NSs also suggested functionalities to record their daily clinical practice activities and to facilitate direct communication among NSs, NPs, and NEs.

#### Technical Architecture

TOPP-N is distributed through official application stores as an application for the iOS and Android systems on digital devices, such computers, tablets, and smartphones. The application can also be accessed at a dedicated TOPP-N webpage [45]. Technical decisions were executed by the application developers only following discussions with the project leader (the first author) after the leader had conferred with the project group.

#### Security, Privacy, and Cost Considerations

The developed prototype is in accordance with the European General Data Protection Regulations of 2018 [46]. All locally stored information is encrypted. The development of the TOPP-N application prototype costed 600,000 Norwegian crowns (US $60,000), excluding salaries and overhead costs.

#### Currently Applied Guidance Model Versus the Guidance Model Supported by TOPP-N

**Figure 8:**
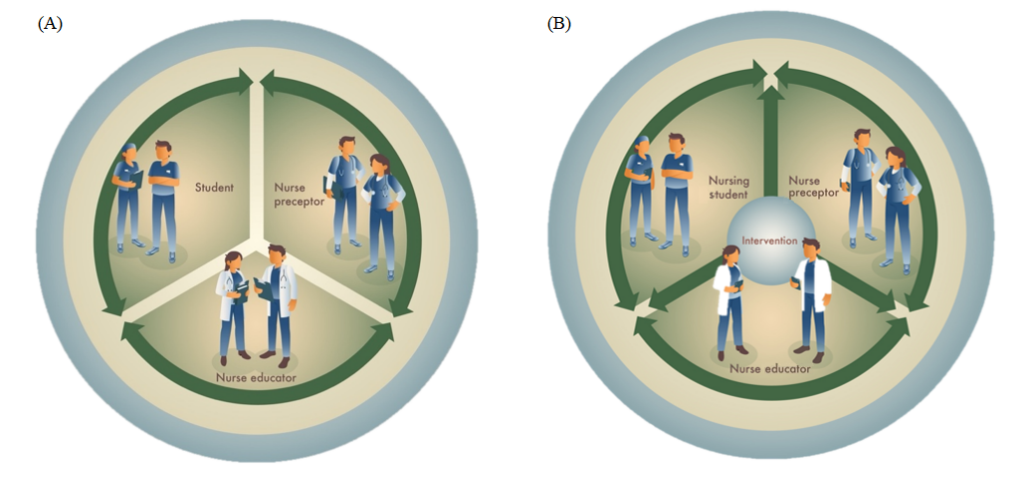
The currently used guidance model (A) and the guidance model supported by the TOPP-N application (B). TOPP-N: Technology-Optimized Practice Process in Nursing.

Figure 8 illustrates how TOPP-N supports the guidance of NSs in clinical practice. The application aims to fill the gaps (address the challenges) that have been identified in clinical practice and to facilitate better integration and interaction among NPs, NEs, and NSs, thus potentially improving cooperation between educational and clinical practice institutions.

## Discussion

### Principal Findings

This research developed a guidance and assessment application prototype (TOPP-N) to support guidance, improve communication, stimulate NSs’ critical thinking, and ensure their structured evaluation in clinical practice. TOPP-N aims to enhance the flexibility, quality, and efficiency of nursing education. The development process combined evidence-based knowledge and user-centered approaches. To our knowledge, this is the first study combining evidence-based knowledge with user input to inform the development of such an application for clinical practice in nursing education. TOPP-N was developed using iterative and inductive processes through the spiral model of application development [30] and was based on (1) contextual inquiry and co-design processes that gathered input from stakeholders (NSs, NPs, and NEs), (2) application content based on the AssCE [40], and (3) application functionality informed by the metacognition process [35] and constructive alignment principles [34].

In Norway, the current options for a guidance model in clinical practice do not include the use of technology. A recent mixed methods systematic review by our research group found a research gap regarding the use of technology-supported guidance models in nursing education worldwide [18]. TOPP-N contributes to closing this gap and addresses the challenges in clinical practice reported by several research studies [7,17,28].

We highlight the importance of user involvement and evidence-based knowledge in developing such an application and show how TOPP-N can address the challenges associated with clinical practice in nursing education from users’ (NSs, NPs, and NEs) perspectives while also meeting the needs of educational institutions, health institutions, and the society.

### Importance of User Involvement

We invited user representatives (NSs, NPs, and NEs) to participate in developing our technology-supported guidance model, an approach that research indicates brings both advantages and challenges [47,48]. One advantage was that our user representatives recognized and confirmed that their challenges in nursing education in clinical practice were identical to those reported in research studies [7,17,28]. Our user representatives also underlined the need for an accessible solution compatible with their existing daily routines that provided positive input and reminders in daily clinical practice. We experienced that the involvement of user representatives was essential to successfully develop a TOPP-N prototype tailored to the challenges associated with clinical practice in nursing education. By underlining the need for intuitive effective functionalities that would not demand too much time, the user representatives also helped us stay focused on user friendliness. When developing the project description, we were aware that a lack of user involvement in the development process could lead to high attrition rates and low adherence in the use of the application. Research suggests that people stop using technologies that do not meet their needs, requirements, or daily routines [25]. Consequently, it was important to incorporate the needs and requirements of the specific user group as highlighted in the workshops. However, we are aware that user involvement may also bring challenges in application development. van Velsen et al [49] pointed out that user involvement in eHealth design is challenging because the few involved users represent only a fraction of the larger user group, so their input may be biased and limited. Those authors [49] also noted that there is often an overreliance on user input.

In this application development, we experienced a challenge related to the inductive process of development. We observed that the user representatives found the concept of a guidance and assessment application to be somewhat abstract and that they had difficulties in formulating specific detailed suggestions for content and functions. This was the main rationale for changing our strategy after the second workshop and presenting a temporary solution with potential design features to the user representatives. This new feedback-based approach stimulated discussion and new suggestions from the stakeholders. Furthermore, after changing the development approach, we found that the stakeholders could now understand the concept of a digital guidance and assessment application and could inductively suggest new functions and improvements. Our experience aligns with the findings of a study that investigated the challenges related to user representatives when taking a co-design approach to developing technological tools (or solutions) [50].

### Evidence-Based Knowledge

One of this study’s main goals was to develop a guidance model that continually stimulates NSs’ cultivation of critical thinking. To meet this goal, we adopted metacognition as the theoretical approach for developing the application’s functionalities. Critical thinking is stimulated through a continuing reflective process that demands self-monitoring and self-correction, and metacognition has been shown to be effective at stimulating critical thinking in pedagogical interventions [51,52]. The application’s workflow drew upon constructive alignment principles [34] to guarantee that the chosen learning activities helped NSs achieve their learning outcomes and to ensure that the assessment criteria were tailored to the expected learning outcomes.

### Quality Assurance in the Learning Process

The TOPP-N application intends to meet the challenges in clinical practice described by several research studies [7,12,17,28] by ensuring good communication between users, structuring the guidance delivered by NPs, and generating an overview of NSs’ clinical practice performance that is available to NSs, NPs, and NEs. In addition, TOPP-N enables NSs to be aware and frequently reminded of the learning outcomes to be achieved in clinical practice by prompting them to plan their day and report their performance daily. Based on the NSs’ reports and the personal daily guidance given through TOPP-N, NPs provide tailored written or verbal feedback that is saved on a server to which NSs and NEs have immediate access. Based on the feedback from NPs, NSs can improve their plan and strategy to achieve the expected learning outcomes for clinical practice, and NEs can support and coach NPs in providing feedback that ensures a pedagogical approach. Another advantage is that, through the application, NPs can always access the expected learning outcomes, enabling them to prioritize the suggestions in the daily guidance to better achieve the goals of clinical practice in nursing education. The described pedagogical process and the application’s documentation of NSs’ clinical practice support quality assurance. To the best of our knowledge, this is the first developed tool that enables documenting the progress of NSs in clinical practice. In 2022, the TOPP-N application received the quality of education award for higher education in Norway awarded by the Ministry of Education and Research (Multimedia Appendix 5).

### Students’ Responsibility for Their Own Learning

Because of the theoretical approach taken in developing its functionality, TOPP-N may stimulate an active learning process and enhance NSs’ responsibility for their own learning. The guidance module allows NSs to choose which learning activities to focus on (see Textbox 1) and to gradually work through the expected learning outcomes at their own pace while being guided by NPs. A recently published study underlined NSs’ need for more guidance [8]. In the evaluation module, the digitalization of the AssCE allows all users (NSs, NPs, and NEs) to prepare for evaluation meetings. The digital evaluation form is available to users from their first day in clinical practice, so NSs can progressively record the finished clinical practice activities that indicate their achievement of learning outcomes and can document their need for further guidance. They can also recall what they have planned and done in the guidance module and use the recorded information and feedback in their self-assessments. Progressively documenting their own development empowers NSs to take command of their own learning, provides a valuable opportunity to establish a basis for the direction of the assessment, and greatly influences the results of the clinical practice evaluation.

### Flexibility and Transferability to Other Professional Education

As mentioned, the expected global nurse shortage must be addressed [4], but solving this problem is challenging, as European Union regulations stipulate that 50% of nursing education include mandatory clinical practice, yet there are not enough clinical placements near educational institutions [53]. This study aimed to develop a guidance application that could be delivered in a technological format supported by several digital platforms, such as tablets, smartphones, and the web, on both Android and iOS systems. TOPP-N enables follow-up and evaluation with ensured guidance quality, making it possible to use several available clinical placements far from educational institutions, including following up with students in exchange programs. Limited number of clinical placements is the major reason why Norwegian educational institutions are not able to increase their education capacity. Using the TOPP-N application, new clinical placements can be used, which will help solve this problem. In addition, by ensuring educational quality and improving the preceptor’s competence, TOPP-N facilitates mentoring tailored to the NSs’ needs. Through this approach, a higher number of students can finish their education within the expected duration, leading to a higher educational effectivity. TOPP-N has been developed to be easily adaptable to other professional education that includes practice and that may face challenges similar to those in nursing education.

### Strengths, Limitations, and Future Directions

This study included a broad range of stakeholders (NSs, NPs, and NEs participating in the project group, as well as researchers and software developers) from the project planning stage through the development process and testing, as recommended by existing research [25]. Mutual learning and shared understanding are core concepts of participatory design, as they ensure mutual respect between stakeholders and enable everyone to take part in the shared decision-making process. Users are not technology experts and do not necessarily have the language to articulate what they need from an application [50]. Consequently, retaining the same sample of users and giving them adequate knowledge of the development process may have made it easier for the stakeholders to participate actively in application development. It was considered important to the development of TOPP-N to solicit user feedback on functionality, layout, and how the material was presented. However, including new user representatives might have added new perspectives in the development process that we were not able to identify.

A further limitation in the application’s development process is that the relevant stakeholders included NSs and NEs only from LDUC, excluding input from other nursing educational institutions in Norway. Another limitation is the use of the AssCE as the basis of the application’s development, which limits the use of other assessment forms. To use TOPP-N, nursing programs need to adopt the AssCE [49]. Further development of TOPP-N will allow educational institutions to include and use their own evaluation forms in the system.

The development was guided by existing development recommendations, and used a broad range of service design methods and a user-centered design approach to facilitate cocreation, mutual learning, and shared understanding among the stakeholders. Although the process followed a participatory design to increase the likelihood of acceptability, usability, and feasibility, these elements will be tested in future studies. In addition to high user involvement and stakeholder input, the development process was guided by well-established theory and concepts from metacognition [35] and constructive alignment [34]. This enhances the future potential to find positive effects.

### Implications for Clinical Practice

TOPP-N is intended to facilitate NSs’ learning processes, ensure quality guidance, and improve communication among NSs, NPs, and NEs. Daily e-reports before and after a shift in clinical practice promote metacognitive strategies that stimulate self-regulated learning and critical thinking. Ongoing feedback is fundamental to NSs’ professional development, as it provides direction and increases their confidence, motivation, and self-esteem. The TOPP-N guidance and assessment application may enhance NPs’ competence in guidance, improve NSs’ learning outcomes in clinical practice, improve the use of resources by enabling remote guidance, and consequently increase nursing education capacity. Better-educated NSs can enhance the quality of care and consequently improve patient safety [54].

### Conclusions

The developed guidance and assessment application will enable NSs to complete daily e-reports, receive feedback from NPs and NEs, and evaluate their learning outcomes in clinical practice based on daily mentoring and documentation. It will also enable remote follow-up by NEs, enabling ongoing support of NSs’ learning progress, and prompt involvement when necessary.

This study offers insights into a user-centered approach for the development of an evidence-based guidance and assessment application to ensure the quality of NSs’ clinical practice, providing a practical example of how a technology-supported guidance model can be developed.

It is important to emphasize that developing an application is a constantly evolving dynamic process, so we must continually focus on further development of its content and function. After a rigorous development process, we believe that the TOPP-N guidance and assessment application prototype is ready to be tested in further intervention studies. The project group has performed follow-up intervention studies to test (usability and feasibility) and examine the effects (randomized controlled trial) of the TOPP-N application. The results will be presented in future research articles.

## Appendix

Multimedia Appendix 1 Final version of the TOPP-N symbol for promotional material. TOPP-N: Technology-Optimized Practice Process in Nursing.

Multimedia Appendix 2 First version of the TOPP-N symbol. TOPP-N: Technology-Optimized Practice Process in Nursing.

Multimedia Appendix 3 First version of the TOPP-N icon. TOPP-N: Technology-Optimized Practice Process in Nursing.

Multimedia Appendix 4 Final version of the TOPP-N icon. TOPP-N: Technology-Optimized Practice Process in Nursing.

Multimedia Appendix 5 Quality of education award for higher education in Norway 2022.

## Abbreviations

AssCE: Assessment of Clinical Education
LDUC: Lovisenberg Diaconal University College
NE: nurse educator
NP: nurse preceptor
NS: nursing student
RN: registered nurse
TOPP-N: Technology-Optimized Practice Process in Nursing
