# Supplementary figures and images for: A Technology-Supported Guidance Model to Increase the Flexibility, Quality, and Efficiency of Nursing Education in Clinical Practice in Norway: Development Study of the TOPP-N Application Prototype
Source: JMIR Hum Factors. 2023 Feb 3;10:e44101. doi: 10.2196/44101 (PMC9938443; doi:10.2196/44101)

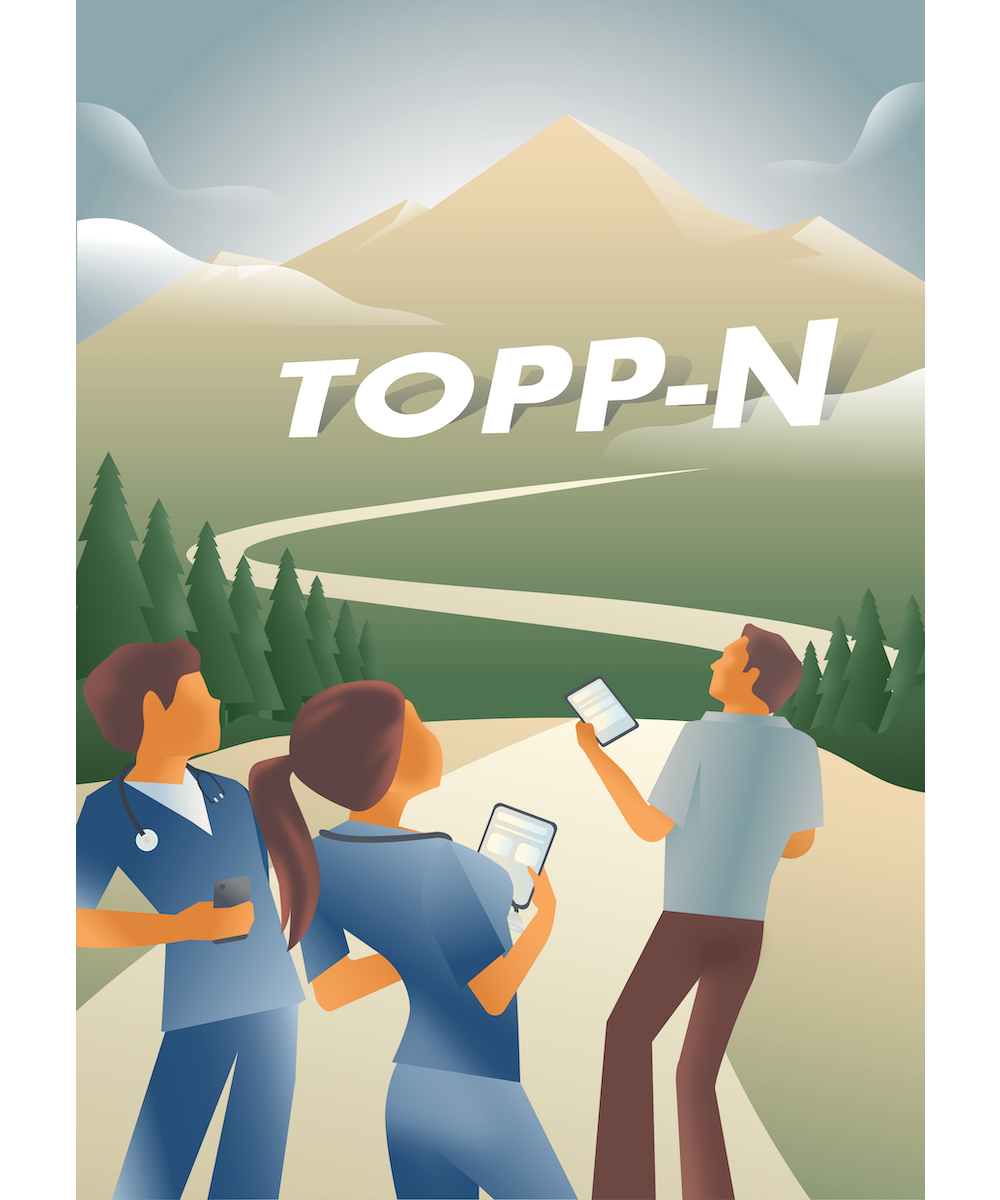

Supplement: Multimedia Appendix 1 [file humanfactors_v10i1e44101_app1.png]

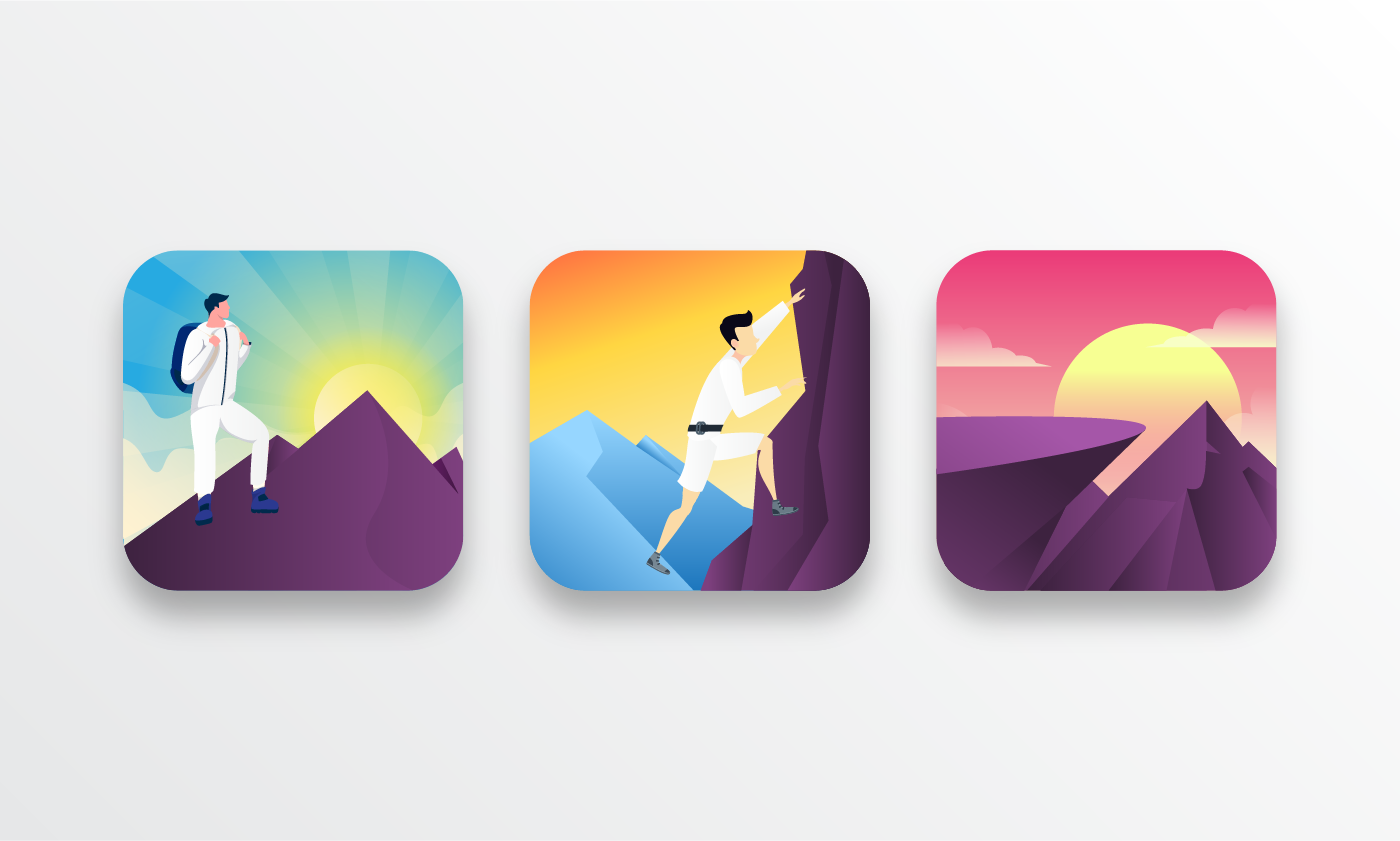

Supplement: Multimedia Appendix 2 [file humanfactors_v10i1e44101_app2.png]

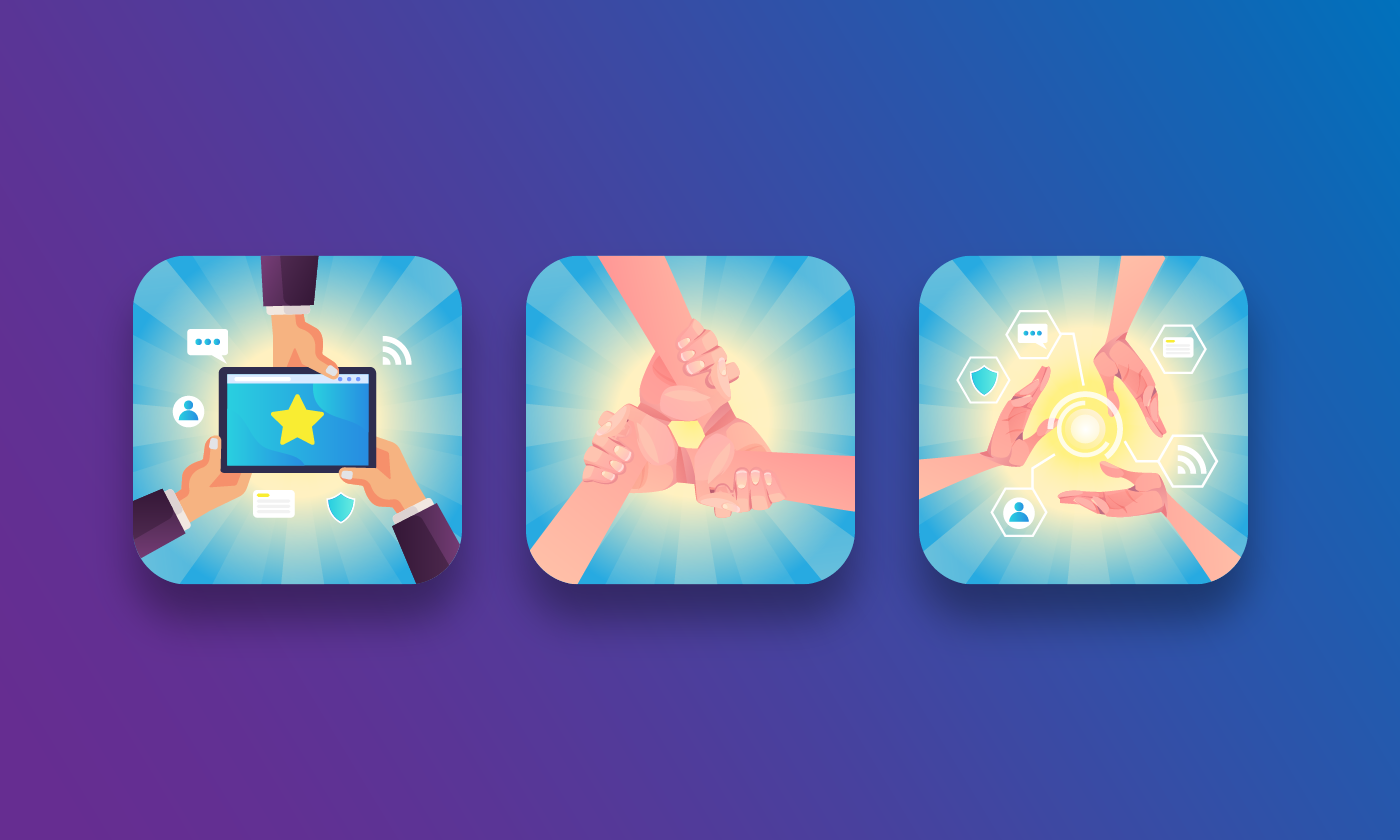

Supplement: Multimedia Appendix 3 [file humanfactors_v10i1e44101_app3.png]

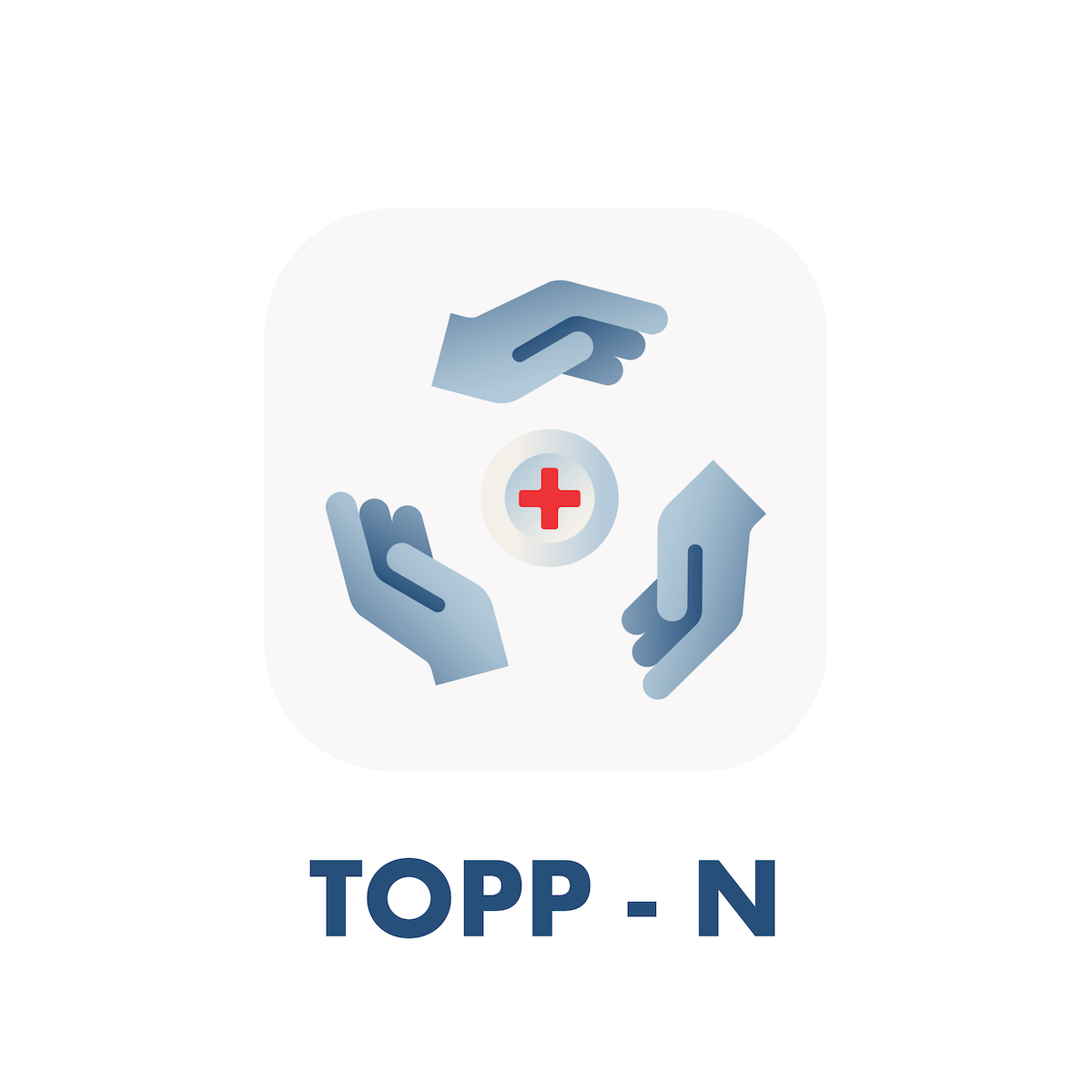

Supplement: Multimedia Appendix 4 [file humanfactors_v10i1e44101_app4.png]

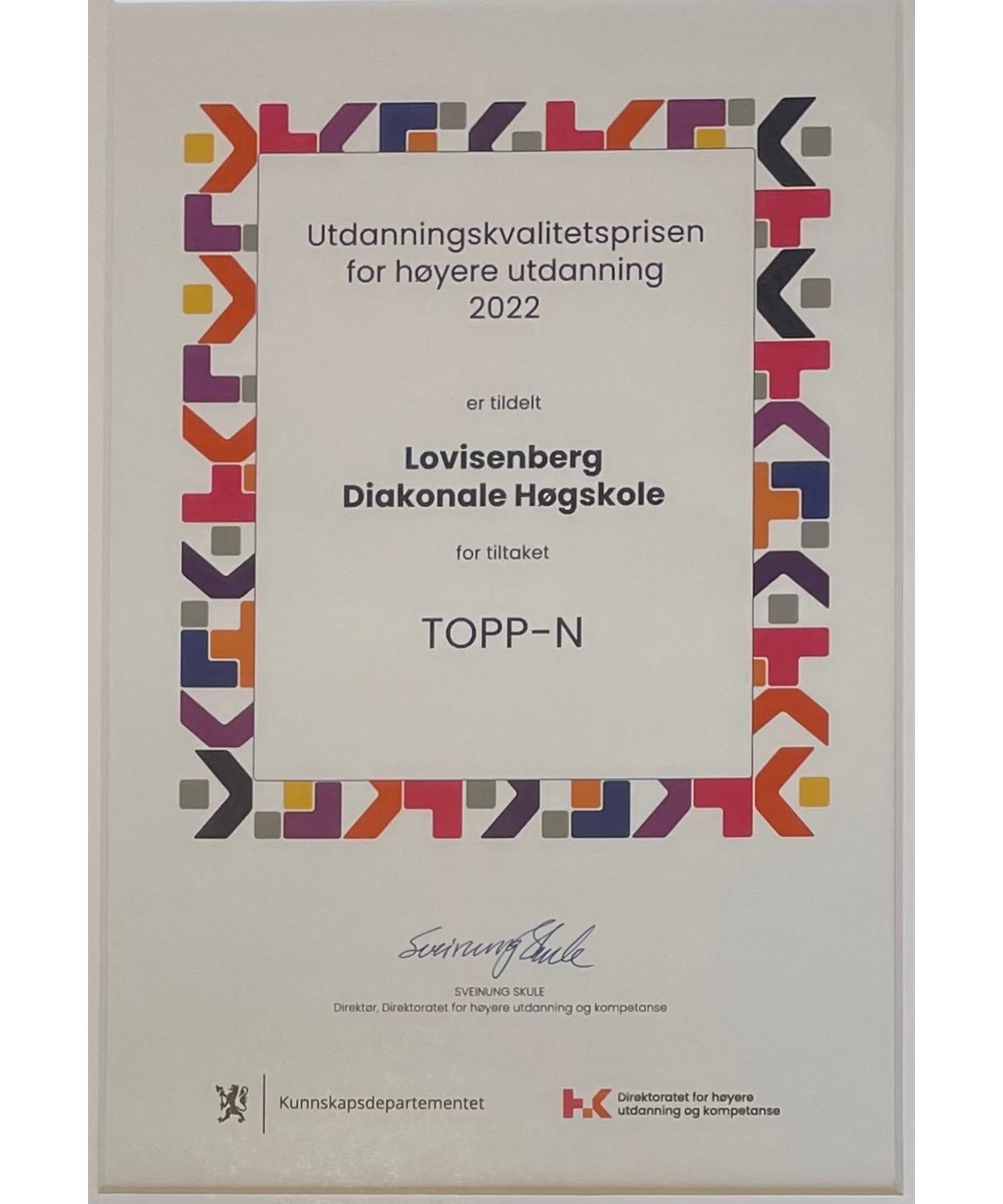

Supplement: Multimedia Appendix 5 [file humanfactors_v10i1e44101_app5.png]
